# Australian men's help-seeking intentions for anxiety symptoms: The impact of masculine norm conformity and gender role conflict

**DOI:** 10.1016/j.heliyon.2024.e29114

**Published:** 2024-04-05

**Authors:** Patrice A. Ford, Carol A. Keane

**Affiliations:** Faculty of Health, School of Allied Health Sciences, Discipline of Psychology, Charles Darwin University, Ellengowan Drive, Casuarina, NT, 0810, Australia

**Keywords:** Australia, Help-seeking, Masculinity, Anxiety, Men, Mental health

## Abstract

Research highlights a discrepancy between the number of men experiencing mental illness and those seeking professional help, particularly for anxiety. Conformity to masculine norms (CMN) and gender role conflict (GRC) have been recognised as barriers to men's mental health help-seeking, but few studies have examined these relationships for anxiety. This study aimed to examine the relationship between anxiety severity and help-seeking intentions in Australian men, and the additional impact of CMN and GRC. A total of 610 Australians aged 18–89 years (*M* = 46.02, *SD* = 17.14) participated in an online survey, which included demographic information and four standardised questionnaires measuring anxiety, help-seeking intentions, CMN, and GRC. CMN but not GRC was found to fully mediate the relationship between anxiety severity and anxiety-related help-seeking intentions. The results have theoretical implications for the study of masculinity and clinical implications for therapeutic approaches for men with anxiety symptoms.

## 1 Introduction

Mental illness is endemic in modern Australia, yet many people who experience symptoms of a mental disorder do not seek professional help. Specifically, national statistics indicate that only 45 % of Australians struggling with their mental health are currently engaged with a professional service; and only a third of this 45 % are men [1]. Men's reduced help-seeking cannot be attributed to better mental health, as indicated by higher rates of substance abuse, violence, and suicidality compared to women [[2], [3]]. Indeed, recent data shows 76 % of total suicides in Australian in 2020 were by men [4]. Depression and substance misuse have been widely studied within the men's mental health literature [[5], [6], [7], [8]]; however, there has been comparatively little research into men's experiences of and help-seeking for anxiety, despite it being the most common mental illness in men [9].

Anxiety is a state characterised by intense, excessive, and persistent fear or worry about everyday situations [10]. Symptoms often include physical sensations, such as rapid heart rate and excessive sweating, and feelings of restlessness, fatigue, and irritability [10]. The relatively little research on men's experiences of anxiety compared to other mental health conditions, such as depression, may be due in part to the well-established association between depression and suicidality [11]. However, anxiety disorders are also potential precursors to suicidality [12,13], further warranting more focused research into men's anxiety.

Within Australia and globally, rates of anxiety diagnosis for men are approximately 50 % less than for women [14,15]. It has been proposed that the differential rates of anxiety between the genders may be an artifact of men under-reporting anxiety-related concerns or neglecting to seek help, and not merely a reflection of lower rates of anxiety in men comparative to women [16]. Several studies evidence that men and adolescent boys are significantly more likely than not to hold stigmatising views about help-seeking for anxiety [[17], [18], [19]]. This same result was found for participants both with and without clinical levels of anxiety symptoms. Therefore, it is unclear at what point – if any – anxiety is deemed severe enough for men to ignore the threat of stigma and seek help. There is evidence from general population studies that psychological symptom severity is associated with *reduced* help-seeking intentions [20,21]. To date, no research has examined the specific relationship between anxiety severity and help-seeking intentions in men.

The mental health help-seeking literature likens help-seeking to a process of adaptive coping, whereby people seek external support to manage internal concerns [22,23]. Where the focus is on gendered or male-specific help-seeking, the impact of masculinity has been widely explored. Conformity to masculine norms (CMN^1^) – defined as the endorsement of socially accepted, traditional male roles and behaviours – has been recognised as a substantial barrier to men's help-seeking [[24], [25], [26]]. Additionally, some research [27,28] has identified negative associations between mental health help-seeking and gender role conflict (GRC^2^), a term used to refer to the emotional and relational distress that can result from rigid adherence to restrictive gender roles [29]. However, most of the research exploring masculinity's impact on men's help-seeking does so in the context of general mental health help-seeking or help-seeking for depressive symptoms [18,19]. There remains a clear need to examine the impact of masculinity on anxiety-specific help-seeking.

### 1.1 Mental health help-seeking

Help-seeking for mental health issues involves communicating the need for personal and emotional support, with the goal of receiving some form of assistance, whether psychological or practical [30]. Mental health help-seeking can be sought from informal sources such as friends and family, or from formal sources such as psychologists, counsellors, or general practitioners [31]. There is strong indication that evidence-based psychological treatment provided by mental health professionals (i.e., formal help-seeking) can greatly aid recovery from psychological illness [[32], [33], [34]].

Within mental health help-seeking literature, help-seeking is generally measured through attitudes, intentions, and behaviours. These three concepts are elements of Azjen's Theory of Planned Behaviour [35], a process model aimed at understanding and predicting behaviour; whereby, attitudes refer to deeply held thoughts and feelings about seeking help, intentions refer to the perceived likelihood of seeking help, and behaviours refer to concrete help-seeking acts (e.g., booking an appointment or meeting with a health professional) [22]. Global research across all three help-seeking domains shows that men have reduced help-seeking compared to women. That is, men demonstrate more negative help-seeking attitudes [6], reduced intention to seek help [36], and fewer help-seeking behaviours [37]. As an identified at-risk group when it comes to mental ill health, the low help-seeking rates of men are a substantial public health concern [38].

Help-seeking attitudes are often the construct in focus within help-seeking research, as attitudes can be easier to operationalise and measure than intentions or behaviours [39]. Further, various studies recognise that men's help-seeking attitudes are positively correlated with intentions to seek help for mental ill health [39,40]. Nonetheless, the extent to which men's help-seeking attitudes *predict* intentions is small, in some cases explaining only 5 % of the total variance in intentions [41]. Research also demonstrates that intentions are more highly correlated with behaviours than are attitudes [42]. Additionally, a recent study investigating the Theory of Planned Behaviour in the context of help-seeking found that intentions strongly and reliably predicted behaviour [43]. Therefore, although attitudes are often used as a proxy for intentions, and subsequently, behaviours, findings indicate that intentions may be a preferrable proxy-measure to attitudes when trying to understand future help-seeking behaviours.

### 1.2 Barries to Men's help-seeking

#### 1.2.1 Conformity to masculine norms (CMN)

It is well established that consideration of the sociocultural construct of masculinity is an imperative within conceptualisations of men's mental health help-seeking [39]. There are various recognised dimensions of masculinity comprising collective practices and attitudes that vary across culture, context, and time [44]. Also termed masculine ‘norms’, these gendered practices and attitudes are socialised rules that generally guide and constrain men's behaviour [44,45]. Western social norms of masculinity hold that men should be independent, stoic, and emotionally controlled; that is, they are encouraged if not expected to act with bravery and conceal any emotion that might portray weakness [46,47]. Given the centrality of emotion identification and expression to mental health help-seeking [48], it is theoretically likely that the emotional control associated with social norms of masculinity constitutes a substantial barrier to men's help-seeking.

Most explorations of the link between CMN and help-seeking have been in the context of general mental health and depression [49], with little to no research directly examining their association in the context of anxiety disorders. Wong and colleagues conducted a large-scale meta-analysis, which found that CMN was consistently associated with unfavourable attitudes toward help-seeking, and the specific masculine norms of emotional control, self-reliance, and power over women were linked to more negative social functioning, depression, and general psychological distress [49]. Other research has reported CMN to be indirectly associated with reduced help-seeking *intentions* via attitudes toward help-seeking [39,50]. Despite the dearth of research examining these relationships within the context of anxiety disorders, there is evidence to suggest these relationships might exist and warrant further examination. For instance, research has revealed that men may perceive themselves as failures if they cannot control experiences of anxiety [11]. Further, males often perceive anxiety-related help-seeking as a personal weakness, with the potential to compromise social status and others’ perceptions of their capabilities as men [17,18]. These examples speak to elements of CMN that may stand in the way of men seeking help for anxiety and highlight a research gap for investigation.

#### 1.2.2 Gender role conflict (GRC)

Related to but distinct from CMN is the concept of masculine gender role conflict (GRC), which refers to the distress that arises from unresolved conflicts between gender role demands and situational expectations [51]. Since GRC was first conceptualised in the early 1980s [52], researchers have considered the extent of the impact gender role socialisation has on men's behaviour and wellbeing [[52], [53], [54]]. The detrimental impact of GRC on social and psychological health is also well-documented [[55], [56], [57]]. Research on the link between GRC and anxiety is somewhat limited. One study found that men with higher levels of GRC also had higher levels of anger, depression, and anxiety [56], and another found GRC was associated with higher social anxiety in young, single men [58]. Some studies have demonstrated a link between GRC and help-seeking attitudes [29,59,60], and another found that GRC was significantly and negatively related to help-seeking attitudes but not at all related to help-seeking intentions [61]. Whether, and to what extent, GRC acts as a barrier for men in seeking help for anxiety-related concerns, remains a gap in current knowledge.

GRC and CMN appear to share a clear theoretical underpinning based on traditional masculinity ideology. Booth suggests men who strongly endorse or enact specified ideals, as in CMN, may consequently lack the flexibility to meet a diverse range of interpersonal and situational expectations, as in GRC [62]. Thus, it is perhaps helpful to conceptualise CMN as the preceding values and characteristics, and GRC as the associated psychological state and behavioural restrictions and patterns [62]. However, despite conceptual links and consistent evidence of a moderate statistical relationship between these masculinity constructs [63], it remains unclear whether their role in help-seeking outcomes is distinct or interdependent.

### 1.3 The present study

The present study aimed to examine the relationship between anxiety severity and help-seeking intentions in male-identifying Australians. Additionally, the study aimed to investigate the impact of CMN and GRC on this relationship to reveal and understand potential barriers to anxiety-specific help-seeking. The following research questions were examined.

1. Does anxiety severity, CMN, and GRC predict help-seeking intentions for anxiety?
2. Does anxiety severity predict CMN?
3. Does anxiety severity predict GRC?
4. Does CMN and GRC, either independently or interdependently, mediate the relationship between anxiety severity and help-seeking intentions for anxiety?

## 2 Method

### 2.1 Participants

An a-priori power analysis indicated that a sample size of 215 was required to have 80 % power for detecting a medium indirect effect size (e.g., standardised beta of 0.30) at *p* < 0.05. To meet eligibility criteria, participants needed to self-identify as male, be 18 years or older, and be an Australian citizen and/or resident. A total of 828 adults at least partially completed the study, of which 217 (26.21 %) participants were excluded from data analysis due to incomplete survey responses, and one additional participant was excluded because they did not identify as male. The final sample consisted of 610 male-identifying adults aged 18–89 years (*M* = 46.02, *SD* = 17.14).

### 2.2 Materials

#### 2.2.1 Demographic items

Participants were asked to report the following demographic details: gender identification, age, birth country, Australian citizen or resident status, years lived in Australia, identification as Aboriginal and/or Torres Strait Islander, primary language spoken at home, sexuality, postcode, employment status, occupation, student status, and highest qualification obtained. Participants were also asked to report whether they had seen a mental health professional in the past 12 months, whether they used alcohol or other substances, and whether they were taking prescription medications.

#### 2.2.2 Help-seeking intentions

The Mental Help Seeking Intention Scale (MHSIS) [64] is a three-item unidimensional measure of help-seeking intentions, adapted from Ajzen's [65] three-item intention instrument. Responses to each of the MHSIS items are rated on distinct 7-point Likert scales, with higher scores on all three items indicating greater help-seeking intentions. The items address the degree to which respondents would intend, try, and plan to seek professional help if experiencing a mental health concern. In the present study, item wording was altered slightly from the original instrument to capture anxiety-specific rather than general mental health concerns. For example, the first item read, “*If I was experiencing anxiety, I would intend to seek help from a mental health professional”*.

In general, intentions are better behavioural predictors than attitudes, as established in the widely accepted Theory of Planned Behaviour [35]. Due to its stronger theoretical underpinnings, the MHSIS was deemed preferrable to some of the more widely used, attitude-based help-seeking measures, such as the Inventory of Attitudes Toward Seeking Mental Health Services (IASMHS) [66]. Although use of the MHSIS is limited, earlier iterations adapted from the same three-item intention instrument have demonstrated sound internal consistency and convergent validity [67,68]. Cronbach's alpha in the present study was 0.96.

#### 2.2.3 Conformity to masculine norms (CMN)

The Conformity to Masculine Norms Inventory–22 (CMNI-22) [47] is a shortened version of the original CMNI developed by Mahalik and colleagues. The scale's purpose is to measure endorsement of traditional masculine gender role norms – specifically, the 11 dominant masculine norms identified in the original scale's development. The CMNI-22 includes 22 items rated on a four-point Likert scale, ranging from 0 (*strongly disagree*) to 4 (*strongly agree*). Nine of the 22 items are reverse scored, and some example items are *“I enjoy taking risks”* and *“I tend to share my feelings”.* The CMNI-22 has shown Cronbach's alphas ranging from 0.70 to 0.75 [[69], [70]]. Cronbach's alpha in the present study was 0.77.

#### 2.2.4 Gender role conflict (GRC)

The Gender Role Conflict Scale Short Form (GRCS-SF) [71] is a revised version of the original GRCS developed by O'Neil and colleagues [72]. The GRCS-SF aims to assess the extent of one's emotional and relational distress resulting from rigid adherence to gender roles and expectations. The measure includes 16 items rated on a six-point Likert scale, ranging from 1 (*strongly disagree*) to 6 (*strongly agree*), with higher scores indicating greater levels of GRC. Some examples of items are *“I do not like to show my emotions to other people”* and “*Winning is a measure of my value and personal worth”*. The GRCS-SF correlates highly with its original version and has demonstrated Cronbach's alphas ranging from 0.77 to 0.80 [[50], [71], [73]]. Cronbach's alpha in the present study was 0.86.

#### 2.2.5 Anxiety severity

The Generalised Anxiety Disorder-7 (GAD-7) [74] is a brief measure used to assess the severity of generalised anxiety over a two-week period. The scale includes seven items rated on a four-point Likert scale, ranging from 0 (*not at all*) to 3 (*nearly every day*). Respondents are required to use the rating scale to indicate how often they have been bothered by various problems over the last two weeks, including *“feeling nervous, anxious, or on edge”* and *“being so restless it's hard to sit still”*.

Although Spitzer and colleagues provide clinical cut-off points [74], the GAD-7 is more often used as a continuous measure of anxiety [75]. Meta-analytic and systematic review research shows that the GAD-7 is psychometrically sounds across general clinical samples, anxiety-specific clinical samples, and community samples [76,77]. Used in community samples, as in the present study, the GAD-7 has consistently shown Cronbach's alphas ranging from 0.85 to 0.89 [[78], [79]]. Cronbach's alpha in the present study was 0.92.

### 2.3 Procedure

Ethics approval for this study was granted by the Charles Darwin University Human Research Ethics Committee (CDU HREC: H21109, valid until December 1, 2022). Participants were recruited through social media advertisements and flyers placed around public spaces (e.g., libraries, sporting facilities, and universities) in the cities of Darwin and Canberra. Social media advertisements were live for a period of 60 days across Facebook, Instagram, and Reddit platforms. Flyer advertisements were also left up for 60 days. To access the survey, participants followed either a link or QR code to the online survey platform, Qualtrics, where they gave informed consent to participate. Participants then provided basic demographic information and completed the four standardised questionnaires.

### 2.4 Data analysis plan

All data screening and analyses were conducted on IBM SPSS Statistics for Mac, version 27 (IBM Corp., Armonk N.Y., USA). An iterative approach was taken to address the four research questions, but before doing so, a multiple linear regression was conducted to ascertain potential demographic covariates impacting anxiety severity. After isolating and accounting for these covariates, a series of multiple regression analyses were conducted to assess research questions one to three. A partial correlation was then conducted to assess the relationship between CMN and GRC, while accounting for the effect of anxiety severity, given these masculinity variables were correlated at *r* = 0.58. As the correlation between residuals remained substantial, research question four was assessed through a serial mediation analysis using PROCESS macro model 6. As mediating variables are treated as consecutive in a serial mediation model [80], the sequencing of the mediators was theoretically determined.

## 3 Results

### 3.1 Data screening

There were no missing data values present in the final sample (N = 610). Visual and statistical inspection of normality plots indicated normal distribution of all variables used in the mediation model. Linear relationships were found between help-seeking intentions and all three dependent variables, and the assumption of no multicollinearity was met, as demonstrated by variance inflation values (VIFs) well below 10 [81]. Finally, a scatterplot of the residuals indicated no violation of homoscedasticity.

### 3.2 Descriptive Statistics

Means, standard deviations, minimum and maximum scores, and possible ranges for the four main variables are reported in Table 1. The broad range of scores on all four standardised measures demonstrated no ceiling or floor effects, which maximises the chances of revealing existing relationships between variables [82].

**Table 1:** Descriptive statistics for variables used in primary analyses.

Variable (measure)	Mean	SD	Minimum	Maximum	Possible range
Anxiety severity (GAD-7)	7.89	6.01	0.00	21.00	0 to 21
Conformity to masculine norms (CMNI-22)	1.17	0.32	0.27	2.41	0 to 4
Gender role conflict (GRCS-SF)	3.05	0.84	1.13	5.63	1 to 6
Help-seeking intentions (MHSIS)	4.14	1.89	1.00	7.00	1 to 7

### 3.3 Data analysis

#### 3.3.1 Determining covariates

A multiple linear regression was first conducted to ascertain covariate impacts of demographic factors on anxiety severity. Results indicated that five of the 12 demographic variables significantly predicted anxiety severity. The variables that significantly and positively predicted anxiety severity were, being a student (*b* = 1.02, *p* = 0.01) and having completed education to at least Year 12 (*b* = 1.61, *p* = 0.02). The demographic variables that significantly and negative predicted anxiety severity were, age (*b* = −0.08, *p* < 0.001), having seen a mental health professional in the past 12 months (*b* = −2.28, *p* < 0.001), and being on prescription medication (*b* = −2.05, *p* < 0.001).

#### 3.3.2 Primary regression analyses

A hierarchical multiple regression was conducted to examine the first research question; do anxiety severity, CMN, and GRC predict help-seeking intentions for anxiety. The demographic covariates identified from the first regression were entered at the first step to account for their impact on anxiety severity. The results of the regression indicated the model was significant at the second step, $R^{2}$ = 0.23, *F*(8, 597) = 22.21, *p* < 0.001. CMN significantly predicted reduced help-seeking intentions (*b* = −1.12, *p* < 0.001), but neither anxiety severity (*b* = −0.02, *p* = 0.17) nor GRC (*b* = −0.17, *p* = 0.15) were significant predictors in either direction.

A second multiple hierarchical regression was then conducted to examine the second research question; does anxiety severity predict CMN. Results indicated anxiety severity significantly predicted CMN, even when accounting for demographic covariates, *b* = 0.01 *p* < 0.001*.* A third and final multiple hierarchical regression was conducted to examine the third research question; does anxiety severity predict GRC. Results indicated anxiety severity also significantly predicted gender role conflict when accounting for demographic covariates, *b* = 0.07 *p* < 0.001.

#### 3.3.3 Mediation analysis

A multiple mediation analysis was conducted to test the final research question; do CMN and GRC mediate the relationship between anxiety severity and help-seeking intentions for anxiety. Firstly, though, to determine whether CMN and GRC played an independent or interdependent mediating role, a partial correlation was carried out on the residuals. The partial correlation showed that CMN and GRC remained correlated after accounting for the effect of anxiety severity, *r* = 0.61, *p* < 0.001. Therefore, a serial multiple mediation analysis was carried out using PROCESS macro model 6, where CMN was determined to linearly precede GRC based on the theoretical patterns outlined in the introduction. Five thousand bootstrap iterations were used in accordance with Hayes’ recommendation [80].

**Fig. 1:**
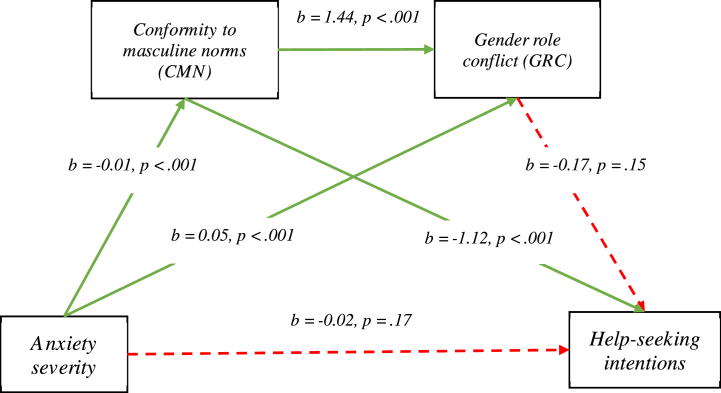
**Unstandardised (b) and standardised (β) coefficients for mediation pathways**. Relationships between variables assessed in the mediation analysis. Statistically significant paths are denoted by green lines and non-significant paths are denoted by red dotted lines. Total effect: *b =* −0.04, 95 % CI [-0.06, −0.01], *β* = −0.12, *p* = 0.002 Direct effect: *b* = −0.02, 95 % CI [-0.05, 0.01], *β* = −0.06, *p* = 0.17 Total indirect effect: *b* = −0.02, 95 % CI [-0.03, −0.01], *β* = −0.06 Indirect effect through CMN: *b* = −0.01, 95 % CI [-0.02, −0.01], *β* = −0.03 Indirect effect through GRC: *b* = −0.01, 95 % CI [-0.02, 0.01], *β* = −0.03 Indirect effect through CMN then GRC: *b* = −0.01, 95 % CI [-0.01, 0.01], *β* = −0.01 Gender role conflict (GRC) Help-seeking intentions. (For interpretation of the references to colour in this figure legend, the reader is referred to the Web version of this article.)

**Fig. 2:**
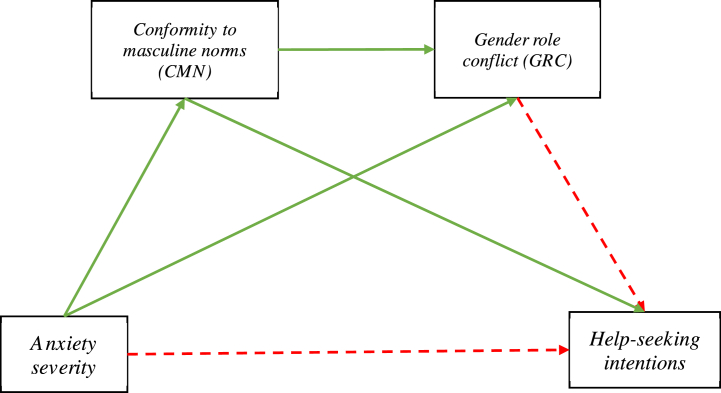
**Relationships between variables assessed in the mediation analysis.** Statistically significant paths are denoted by green lines and non-significant paths are denoted by red dotted lines. Total effect: *b =* −0.04, 95 % CI [-0.06, −0.01], *β* = −0.12, *p* = 0.002 Direct effect: *b* = −0.02, 95 % CI [-0.05, 0.01], *β* = −0.06, *p* = 0.17 Gender role conflict (GRC) Help-seeking intentions Conformity to masculine norms (CMN) Anxiety severity. (For interpretation of the references to colour in this figure legend, the reader is referred to the Web version of this article.)

The results of the serial mediation indicated that CMN, but not GRC, fully and independently mediated the relationship between anxiety severity and reduced help-seeking intentions for anxiety. Importantly, having seen a mental health professional in the past 12 months was a covariate for all mediation paths involving CMN. The total indirect effect of the mediation model was significant, *b* = −0.02, 95 % CI [−0.03, −0.01]. A significant and negative indirect effect was found for anxiety severity on help-seeking through CMN, *b* = −0.01, 95 % CI [−0.02, −0.01] but not through GRC, *b* = −0.01, 95 % CI [−0.02, 0.01]. The indirect effect of anxiety severity on help-seeking, serially, through CMN then GRC was also not significant, *b* = −0.01, 95 % CI [−0.01, 0.01]. While the direct effect of anxiety severity on help-seeking intentions was not significant in the mediation model, the total effect of this relationship was significant. These findings indicate that anxiety severity directly impacts help-seeking intentions in isolation of the other variables included in the model; this is reflected in the non-significant relationship between these variables in the hierarchical regression, which was modelled to include CMN and GRC. The relationships revealed in the mediation analysis are presented in Fig. 1 (detailed) and Fig. 2 (simplified).

## 4 Discussion and conclusions

The present study examined relationships between anxiety severity, help-seeking intentions for anxiety symptoms, and the masculinity constructs of conformity to masculine norms (CMN) and gender role conflict (GRC). The primary aim was to investigate the impact of CMN and GRC on the relationship between anxiety severity and help-seeking intentions in male-identifying Australians, to reveal potential barriers to anxiety-specific help-seeking. Consistent with previous work [17,18,39], our results showed that higher CMN predicted men's reduced help-seeking intentions for anxiety. Conversely, we found no evidence for GRC's implication in anxiety-related help-seeking, despite evidence of an association in previous research [61]. Admittedly, Nagai found a negative relationship only between GRC and help-seeking attitudes but not intentions [61]. In the present study, anxiety severity predicted reduced help-seeking intentions for anxiety; however, this relationship did not remain when masculinity factors were considered.

The present study also found positive predictive relationships between anxiety severity and both masculinity constructs. These results are consistent with previous research linking higher CMN and GRC with poorer mental health outcomes and increased anxiety [49,58]. Finally, the primary novel finding of this study was that higher CMN, but not GRC, fully and independently mediated the relationship between men's anxiety severity and reduced anxiety-related help-seeking intentions. Although our study is the first to have investigated this specific pattern of relationships, our overall findings appear broadly consistent with theoretical and empirical suggestions within the literature; namely, that ideologies of traditional masculinity (a) leave men vulnerable to experiencing anxiety, and (b) constitute substantial barriers to anxiety-related help-seeking.

### 4.1 Theoretical interpretations and implications

The findings of the present study have important theoretical and research implications, in particular for conceptualisation of masculinity within the mental health domain. A conceptual shift within the literature over the past two decades, from *traditional masculinity* to *progressive masculinity,* has aimed to capture the diverse, complex, and fluid nature of modern men's experiences [83,84]. Despite this conceptual shift, the most widely utilised and validated measures in the psychology of masculinity literature remain founded on traditional masculinity (e.g., CMNI-22; GRCS-SF). This limitation was noted from the outset of this research; however, although the attitudes, values, and practices reflected in these measures may no longer constitute ideal or even socially acceptable male norms, our findings suggest there remains value in studying their impact in modern Australia. Relatedly, Australian research suggests that, as dominant masculinity ideals shift, men must navigate a series of contradictory requirements to maintain socially-acceptable gender expression [85]. As the literature on progressive masculinity develops, there will be value in exploring whether the more complex expectations of modern masculinity reduce men's help-seeking intentions further.

Findings from the present study also emphasise the importance of differentiating between and considering all aspects of men's experiences of masculinity. Specifically, our finding that CMN but not GRC predicted reduced help-seeking intentions suggests that the endorsement of traditionally masculine norms, compared with the intrapersonal conflict sometimes facilitated by that endorsement, may be more pertinent to men's intentions to engage with professional services. The link between CMN and GRC has not been explored extensively within the literature. Nevertheless, much of the existing research implies theoretical similarities based on shared ideologies of traditional masculinity [62,63,86]. The implication of our findings for the continued study of CMN and GRC is that, while the constructs are highly associated beyond the shared impact of anxiety severity, they likely have distinct social, behavioural, and psychological consequences.

Previous research has indicated relationships between psychology symptom severity and reduced help-seeking intentions [21,87]. The present study mirrored this pattern, with anxiety severity directly predicting anxiety-specific help-seeking; however, this was only the case when the impact of masculinity variables on this relationship was not considered. It is also worth noting that previous research linking increased symptom severity to reduced help-seeking was specific neither to men nor anxiety. The present study was the first to examine and find support for CMN's mediating role in the relationship between anxiety severity and reduced help-seeking intentions for anxiety. Thus, there is now evidence that the endorsement of traditional male role norms drives reduced help-seeking intentions in Australian men with higher levels of anxiety. One potential explanation for this is that higher levels of anxiety are experienced with particular shame by men who strongly endorse masculine norms. Although there is currently no research linking men's experiences of anxiety to feelings of shame, there is evidence that shame plays a prominent role in men's experiences of depression [8] and post-traumatic stress disorder [88], as well as their propensity to seek help for intimate partner abuse [89]. Similarly, regarding the findings of the present study, perhaps Australian men who strongly endorse masculine norms are less likely to seek help for anxiety because such symptoms are at odds with norms of self-sufficiency, taking charge, and exerting emotional control.

### 4.2 Clinical implications

This study's findings are also important clinically**.** Our results suggest it is unlikely men with high CMN and clinical levels of anxiety will present for treatment in clinical settings. Nevertheless, the present study suggests there may be value in considering norms of self-reliance and emotional control when working with male clients in contexts where they have not self-referred for their mental health (e.g., in correctional facilities and hospitals). Addressing, or at least discussing, masculine norms therapeutically in such contexts may assist clients to speak more openly about potential anxiety-related concerns. Novak and colleagues suggest that openly discussing men's subjective barriers to help-seeking may be useful within therapeutic and health-care contexts [90]. It might also be worthwhile incorporating a focus on masculine norms and expectations into standard-practice cognitive intervention for men with anxiety, as has been suggested in previous research [45]. Further research is needed to assess the usefulness of such therapeutic approaches.

### 4.3 Strengths and limitations

The primary strength of the present study is that it was the first to examine the role of masculinity in men's help-seeking intentions for anxiety symptoms in an Australian population. Despite the prominent impact of anxiety in the lives of Australian men, such research has been absent until now. Another factor setting this study apart from previous research in this area is the explicit inclusion of male-identifying Australians who were not necessarily born male. As social norms around gender identities vary and develop – as they have done so prominently over the past decade [91] – it is important that mental health research reflects these shifts. In line with changes in the masculinity discourse toward a progressive masculinity framework [92], the present study measured a diverse range of men's experiences to capture the state of men's mental health in Australia, with the goal of making actionable change.

Despite its novelty and strengths, the present study needs to be interpreted in the context of its limitations. Firstly, romantic relationship- or partnership-status is a potential confound in this study. Given that traditional masculinity ideals have the potential to be considered sexist and homophobic by modern standards, higher CMN and GRC may be associated with lack of personal support in the form of intimate partnerships. This is particularly salient for the present study, as being in a relationship has been linked to better mental wellbeing, particularly for men [93]. Future research would benefit from including a question on relationship status in their research survey, to reveal and then control for existing relationships as necessary.

The current study's findings may be limited by its sample. As the majority of participants were recruited through social media, there is potential for socioeconomic and cultural bias to limit the sample's representation of a diverse Australian population. Further, the measure used to assess anxiety severity, the GAD-7 [74], only captures generalised anxiety symptoms. For a more comprehensive investigation of masculinity's impact on anxiety and associated help-seeking, future research should measure men's experiences of other common anxiety disorders, such as panic disorder and social anxiety. Similarly, further specificity regarding masculine norms would be a useful focus for future research. For example, it would be helpful to isolate norms of self-sufficiency from emotional restriction to better understand the mechanisms through which masculine adherence facilitates reduced help-seeking intentions.

Interpretations of the present study's findings are limited to impacts on help-seeking *intentions*. Although there is evidence to suggest the Theory of Planned Behaviour holds in relation to help-seeking (i.e., help-seeking intentions predict active help-seeking behaviours [42,43]), we cannot assume this is the case for men or anxiety-specific help-seeking. It is also possible that our study found no predictive relationship between GRC and help-seeking intentions because GRC likely constitutes the psychological and behavioural consequences of CMN [62], and therefore might be more closely linked to functional outcomes. By incorporating a measure of anxiety-related help-seeking *behaviour*, future research might reveal an existing relationship with GRC.

### 4.4 Future directions

The aforementioned limitations should all be addressed in future research to provide a stronger picture of the barriers to men's help-seeking for anxiety. Crucially, substantial changes also must be made to the way researchers operationalise traditional masculinity moving forward. The present study has highlighted the ongoing need to examine aspects of traditional masculinity, even with emerging discourse around progressive masculinity; however, the measures used here to capture traditional masculinity are rapidly becoming outdated in the context of modern Australia. It is vital that the prevailing components of traditional masculinity continue to be studied within men's mental health research, but with modern measures that both reflect and welcome the diverse experiences of Australian men. Alongside the development of more appropriate survey measures, future studies should incorporate qualitative and mixed research designs to gain richer accounts of men's experiences of their masculinity and subsequent the impacts on mental health and help-seeking. Finally, it may be valuable for future research to elucidate the nature and direction of anxiety's link to masculinity.

### 4.5 Conclusion

This research claims the novel finding that CMN fully mediates the relationship between anxiety severity and reduced help-seeking intentions for anxiety in a general population sample of male-identifying Australians. Furthermore, results highlight predictive relationships between anxiety severity and the two masculinity constructs of CMN and GRC. Together, these findings contribute to the growing research body on men's formal mental health help-seeking intentions. This study has important theoretic implications for the future research into men's mental health and masculinity within the behavioural sciences. The findings can also be used to inform therapeutic intervention approaches for men presenting with anxiety or other concerns. Future research should focus on exploring the specific masculine role norms implicated in help-seeking reluctance and anxiety severity, as well as considering more specific anxiety presentations and in the context of explicit help-seeking behaviours. Finally, there is a strong need to update measures of traditional masculinity to better capture the diverse experiences of modern Australian men.

## Data availability statement

Deidentified data can be provided on an email request to the corresponding author.

## CRediT authorship contribution statement

**Patrice A. Ford:** Investigation, Formal analysis, Data curation, Conceptualization, Methodology, Writing – original draft. **Carol A. Keane:** Writing – review & editing, Supervision, Methodology, Formal analysis, Conceptualization.

## Declaration of competing interest

The authors declare that they have no known competing financial interests or personal relationships that could have appeared to influence the work reported in this paper.

## Footnotes

1 Conformity to masculine norms.

2 Gender role conflict.
